# Thrombotic Thrombocytopenic Purpura After Ad6.COV2.S Vaccination

**DOI:** 10.7759/cureus.28592

**Published:** 2022-08-30

**Authors:** Sruthi Ramanan, Harjinder Singh, Priya Menon, Parth M Patel, Vrajesh Parmar, Devin Malik

**Affiliations:** 1 Internal Medicine, Henry Ford Health System, Jackson, USA; 2 Medicine/Surgery, Saveetha Medical College, Chennai, IND; 3 Hematology/Oncology, Henry Ford Health System, Jackson, USA

**Keywords:** ad6.cov2.s, vaccination, covid-19, vitt, ttp

## Abstract

Thrombotic thrombocytopenic purpura (TTP) is caused by the deficiency of ADAMTS13, a von Willebrand factor cleaving protease, which results in thrombotic microangiopathy. It is characterized by microangiopathic hemolytic anemia, thrombocytopenia, and microvascular thrombosis leading to organ damage. It has an extremely high mortality rate if left untreated, making early diagnosis and treatment of the utmost importance. We report a case of TTP that developed after vaccination with Ad26.COV2.S COVID vaccine.

We present a case of a 50-year-old African American female who presented with dyspnea one week after receiving the first dose of Ad26.COV2.S vaccine. Initial labs showed anemia, thrombocytopenia, and markers of intravascular hemolysis. The suspicion for thrombotic thrombocytopenic syndromes (TTS), vaccine-induced thrombotic thrombocytopenia (VITT), TTP, and Immune thrombocytopenic purpura (ITP) was high based on the history and laboratory results. Computed tomography (CT) of the chest and ultrasound of bilateral lower extremities did not show any evidence of thrombosis. The absence of thrombosis in the presence of a high PLASMIC score increased the suspicion of TTP over the other differentials. Diagnosis of TTP was confirmed when the ADAMTS13 level was low with an elevated autoantibody inhibitor level. The patient underwent treatment with corticosteroids, plasmapheresis, and rituximab with improvement in symptoms and platelet count.

TTP and VITT are the possible differential diagnosis for a patient presenting with anemia, thrombocytopenia, and signs of hemolysis after vaccination with Ad26.COV2.S. It is necessary to differentiate these two clinical entities as the management varies based on the diagnosis.

## Introduction

Thrombotic thrombocytopenic purpura (TTP) is a rare, life-threatening disease with an incidence of approximately two persons per million per year. It is characterized by a severe deficiency of ADAMTS13, which is a von Willebrand factor cleaving protease. The deficiency of ADAMTS13 leads to the formation of platelet-rich thrombi in the microvasculature [1]. The thrombi can lead to the development of TTP which is classically characterized by the pentad of fever, hemolytic anemia, thrombocytopenia, and renal and neurologic dysfunction [2]. There have been multiple reports citing the association of TTP with recent immunization with COVID-19 vaccines, including the BNT162b2 COVID-19 vaccine [3]. There have been reports of cerebral venous sinus thrombosis and thrombocytopenia in patients receiving the Ad26.COV2.S Johnson & Johnson (JJ) vaccine [4]. A new clinical syndrome characterized by thrombosis at atypical sites combined with thrombocytopenia has been observed in multiple patients, days after vaccination with the ChAdOx1 nCoV-19 vaccine [5], and similar clinical sequelae were reported in patients after vaccination with the Ad26.COV2.S vaccine [6]. TTP may occur after other vaccinations such as influenza, pneumococcal, and H1N1 [7-9].

## Case presentation

The patient is a 50-year-old African American female with a medical history of type 2 diabetes mellitus, hypertension, chronic obstructive pulmonary disease, poly-substance use disorder including cocaine use, and bipolar disorder who presented to the emergency department with a two-day history of shortness of breath. She denied fevers, chills, nausea, cough, sputum production, chest pain, palpitations, leg swelling or pain, bleeding, bruising, hematuria, rash, headaches, and focal weakness. She received the first dose of Ad26.COV2.S COVID vaccine one week prior to her presentation. She did not have any other medical conditions. Physical examination showed normal vital signs, mild-respiratory distress, and normal breath sounds, and abdominal examination was unremarkable. Clinical suspicion for infections was low as she was hemodynamically stable and a focus of infection was identified. The initial labs are shown in Table 1.

**Table 1:** Initial labs of the patient on presentation Hb: hemoglobin; Hct: hematocrit; Plt: platelets; WBC count: white blood cell count; LDH: lactate dehydrogenase; BUN: blood urea nitrogen; CRP: c-reactive protein; ESR: erythrocyte sedimentation rate

Lab	Value	Normal range
Hb	6.5 mg/dl	12-15 mg/dl
Hct	20.4	36-46
Retic percent	5.1%	0.5-1.5%
Ferritin	603 ng/ml	11-307 ng/ml
Plt	11,000/μl	150,000-450,000/μl
WBC count	9.4K/μl	3.8-10.6 K/μl
LDH	1126 IU/l	100-220 IU/l
Haptoglobin	30 mg/dl	30-300 mg/dl
Total bilirubin	1.8 mg/dl	<1.2 mg/dl
Direct bilirubin	0.4 mg/dl	0-0.3 mg/dl
BUN	17 mg/dl	10-25 mg/dl
Creatinine	0.8 mg/dl	<1.03 mg/dl
CRP	4.9 mg/dl	<0.5 mg/dl
ESR	78 mm/h	0-20 mm/h
D-dimer	9.18 μg/ml	<0.5 μg/ml

A peripheral blood smear showed schistocytes. The patient's presentation and laboratory findings were consistent with hemolytic anemia. The ultrasound of bilateral lower extremities and CT chest did not show any evidence of thrombosis.

Given the exigency of the condition, the patient was suspected to have TTP. The patient’s PLASMIC score (prediction tool for diagnosis and treatment of TTP) was 7, this put the patient at considerable risk for severe ADAMTS13 deficiency (<15% activity level), prompting urgent plasmapheresis. Unfortunately, caplacizumab, a drug that prevents platelet aggregation by inhibiting the interaction between vWF and glycoprotein Ib receptor, was not on the formulary, and thus it was not administered before the plasma exchange. 

The patient received three cycles of plasma exchange along with a dose of rituximab. She also got high-dose steroids in the form of methyl prednisone 125 mg twice a day for five days. The patient's platelet counts slowly started to recover during this time (Table 2). The patient's ADAMTS 13 level came out to be <10% with an elevated ADAMTS13 inhibitor level which suggested an acquired cause for the TTP. The patient was discharged after three days of plasma exchange; she was maintained on a steroid taper and followed up with outpatient hematology to complete a total of five rituximab infusions. 

**Table 2:** Monitored trends in daily labs (days 0-4) LDH: lactate dehydrogenase

Lab	Day 0	Day 1	Day 2	Day 3	Day 4
Platelet count (K/μl)	10	39	73	126	188
LDH (IU/l)	1129	741	401	358	296
Bilirubin (mg/dl)	1.8	0.7	0.4	0.4	0.2
Fibrinogen (mg/dl)	446	250	-	-	-

## Discussion

TTP is a rare condition and can rapidly lead to a fatal outcome in the absence of treatment, raising multiple diagnostic and therapeutic challenges [10]. A distinct entity known as vaccine-induced TTP (VITT) has been described by the American Society of Hematology, which requires the presence of all five markers: COVID vaccine four to 42 days before symptom onset, any venous or arterial thrombosis (often cerebral or abdominal), thrombocytopenia (platelet count less than 150 × 10^9^/l), positive platelet factor 4 heparin-induced thrombocytopenia (PF4 HIT) enzyme-linked immunosorbent assay (ELISA), markedly elevated D-dimer (> 4 times the upper limit of normal). 

In our patient, there was no known venous or arterial thrombosis. Hence, this patient does not fit into the description of VITT. This patient had laboratory-confirmed TTP one week after Ad26.COV2.S vaccine. This case provides an understanding of the possibility of having TTP after vaccinations, which may not necessarily be VITT. It is necessary to differentiate these two entities as the management differs significantly. 

VITT is treated with intravenous immunoglobulin (IVIG) and non-heparin anticoagulation. The presence of PF4 antibodies in VITT leads to platelet activation and increased thrombosis in similar pathophysiology as heparin-induced thrombocytopenia and is hence treated with non-heparin anticoagulation [11]. On the other hand, TTP is treated with intravenous steroids, plasma exchange, and immunotherapeutic agents such as caplacizumab, and rituximab [12]. Moreover, anticoagulation agents, the mainstay of treatment in VITT, are ineffective in TTP.

## Conclusions

TTP and VITT are the possible differential diagnosis for a patient presenting with anemia, thrombocytopenia, and markers of hemolysis after vaccination with Ad26.COV2.S. Differentiating these two conditions is necessary as the management varies significantly.

## References

[1] Chiasakul T, Cuker A (2018). Clinical and laboratory diagnosis of TTP: an integrated approach. Hematology Am Soc Hematol Educ Program.

[2] Stanley M, Killeen RB, Michalski JM (2022). Thrombotic thrombocytopenic purpura. StatPearls [Internet].

[3] Alislambouli M, Veras Victoria A, Matta J, Yin F (2022). Acquired thrombotic thrombocytopenic purpura following Pfizer COVID-19 vaccination. EJHaem.

[4] See I, Su JR, Lale A (2021). US case reports of cerebral venous sinus thrombosis with thrombocytopenia after Ad26.COV2.S vaccination, March 2 to April 21. JAMA.

[5] Greinacher A, Thiele T, Warkentin TE, Weisser K, Kyrle PA, Eichinger S (2021). Thrombotic thrombocytopenia after ChAdOx1 nCov-19 vaccination. N Engl J Med.

[6] Muir KL, Kallam A, Koepsell SA, Gundabolu K (2021). Thrombotic thrombocytopenia after Ad26.COV2.S vaccination. N Engl J Med.

[7] Dias PJ, Gopal S (2009). Refractory thrombotic thrombocytopenic purpura following influenza vaccination. Anaesthesia.

[8] Kojima Y, Ohashi H, Nakamura T (2014). Acute thrombotic thrombocytopenic purpura after pneumococcal vaccination. Blood Coagul Fibrinolysis.

[9] Hermann R, Pfeil A, Busch M (2010). Very severe thrombotic thrombocytopenic purpura (TTP) after H1N1 vaccination. [Article in German]. Med Klin (Munich).

[10] Coppo P, Cuker A, George JN (2019). Thrombotic thrombocytopenic purpura: toward targeted therapy and precision medicine. Res Pract Thromb Haemost.

[11] Bussel Bussel, J. B. (2021, December 17 (2022). Vaccine-induced immune thrombotic thrombocytopenia. https://www.hematology.org/covid-19/vaccine-induced-immune-thrombotic-thrombocytopenia.

[12] Potti A, Ramiah V, Ortel TL (2005). Thrombophilia and thrombosis in thrombotic thrombocytopenic purpura. Semin Thromb Hemost.

